# Stress, Burnout, and General Mental Health among Healthcare Workers in Poland during the Long-Lasting COVID-19 Pandemic

**DOI:** 10.3390/healthcare11192617

**Published:** 2023-09-24

**Authors:** Natalia Budzyńska, Joanna Moryś

**Affiliations:** Department of Clinical Psychology, Faculty of Health Sciences, Medical University of Gdańsk, 80-210 Gdańsk, Poland

**Keywords:** mental health, burnout, stress, healthcare professionals, COVID-19, SARS-CoV-2

## Abstract

Medical professions are characterized by a great responsibility for human health and life; they are also vulnerable to burnout. The outbreak of the COVID-19 pandemic has brought new challenges and threats. This study aimed to assess the mental health of healthcare workers after a year and a half of working in COVID-19 pandemic conditions. The Perceived Stress Scale (PSS-10), the Link Burnout Questionnaire (LBQ), and the General Health Questionnaire (GHQ-28) were utilized in this cross-sectional investigation. A total of 335 healthcare employees from Polish hospitals (median age 44 years) filled out online questionnaires between the 16 August 2021 and the 30 March 2022. Most of the sample was female (86%). In this study, 40.0% of the surveyed healthcare workers reported a high stress intensity. Burnout was reported by 9.6% of the workers, and the most frequently experienced symptom was psychophysical exhaustion. Almost half of the healthcare workers surveyed (49.6%) reported health disorders at both the mental and physiological levels. Interestingly, working in a COVID-19 ward did not significantly differentiate healthcare workers in any of the evaluated variables: PSS-10 (gr. A F = 1.21; gr. B F = 0.71; *p* > 0.05), LBQ (gr. A F = 1.89, F = 0.94, F = 1.08, F = 2.57; gr. B F = 0.32, F = 1.14, F = 0.77, F = 0.36; *p* > 0.05), and GHQ-28 (gr. A F = 0.85, F = 0.52, F = 0.57, F = 0.31; gr. B F = 0.31, F = 0.06, F = 0.06, F = 0.54; *p* > 0.05). Furthermore, there were no statistically significant differences between the compared occupational groups of healthcare workers: PSS-10 (F = 1.08; *p* > 0.05) and GHQ-28 (F = 1.78; F = 0.85; F = 0.62; F = 0.54; *p* > 0.05). The mental health of healthcare workers is alarming, and psychophysical conditions can affect the quality of work and relations with patients. Psychological care in workplaces and workshops that build resources for dealing with difficult situations are necessary.

## 1. Introduction

Understanding the overall features and working conditions of medical professions is crucial in order to fully grasp the burdens experienced by healthcare workers during the COVID-19 pandemic. It is well known that medical professions are at high risk of burnout [1,2], and the outbreak of the pandemic carries additional risks for the mental health of healthcare workers. Numerous studies demonstrate that medical professionals are vulnerable to the pandemic’s harmful mental health repercussions [3,4,5].

People working in medical professions have a huge responsibility for human health and life. These occupations are distinguished by a quick pace of work, shift work, lengthy shifts, the requirement to be on call duty, and work under pressure and stress, as well as [1,5,6] interacting with human tragedies and witnessing pain and suffering [7]. While working conditions and the organization of healthcare systems vary across countries, unfortunately, many national healthcare systems are struggling with staff shortages. As a consequence, the sector is often accompanied by overtime, a fast pace of work, understaffing, and, therefore, work overload, equipment shortages, and low wages [1]. These factors can have a huge impact on the well-being of healthcare workers, including chronic stress and burnout [8,9]. Other factors that affect how work is organized itself, such as the relationships with superiors and coworkers, a lack of participation in decision-making, the environment at work, the pressure from superiors, and even mobbing, all increase the risk of burnout syndrome [9,10]. In addition, individual factors are also crucial, including one’s personality traits [11], cognitive and social resources, external locus of control, passive ways of dealing with difficult situations, or the confrontation of values and ideas about work with the reality found in the workplace [9]. Depending on the measurement tool used and the criteria adopted, between 32% and 50% of healthcare workers experience burnout [7,10,12], yet rates in Poland are higher, with up to 67% of medics experiencing burnout [12]. It is particularly concerning that young medics starting their professional careers are the most at-risk of burnout [9,13,14], considering that the average age of a nurse in Poland is 52 years [13]. Moreover, research indicates that up to half of healthcare workers may experience high levels of stress [8].

At the beginning of 2020, healthcare systems globally experienced further strain with the emergence of SARS-CoV-2. On the 11 March 2020, the SARS-CoV-2 virus appeared in Wuhan, China; in December 2019, with the very rapid spread of the SARS-CoV-2 virus around the world, the WHO declared a global pandemic [15,16]. By the 18 July 2023, 767,972,961 people worldwide had contracted COVID-19 (WHO, accessed on 18 July 2023). In Poland, by the 18 July 2023, 6,518,036 people had been infected (200,000 of whom were medics) [16,17]. To limit the spread of COVID-19, governments around the world introduced various types of restrictions and lockdowns [16]. At the same time, protective measures were introduced, such as the obligation to wear masks in public places [16]. In Polish hospitals, scheduled hospital admissions and treatments were suspended, with only those necessary to save lives being performed [16]. At that time, medics had to face not only a new infectious disease but also the need to use additional personal protective equipment and its shortages, as well as their fear for their health and the health of their loved ones. Many of them lived separately so as not to risk transmitting the infection to family members [18,19,20,21]. Some medics were transferred to work in COVID-19 wards, resulting in a break in practice in their area of specialization or a lack of development opportunities, as well as a greater feeling of pressure [22]. During the pandemic, medics worldwide were seen as heroes, including in Poland. As a token of thanks and admiration for healthcare workers, many people could be seen clapping from their balconies at a certain time of day [23]. At the same time, due to a fear of the new virus, many medics experienced social ostracism [18] and were intimidated, challenged, or threatened; indeed, there were reported cases of vandalism such as damaged tires [16,24,25]. In addition, fake news and conspiracy theories about the virus and subsequent vaccines were spreading around the same time, and there was a lack of trust in scientists and healthcare workers among some people [26].

As described above, the situation was difficult for healthcare workers even before the pandemic. The working conditions during the pandemic placed additional burdens on health systems and, above all, on healthcare workers around the world. To this should be added the context of work in Polish hospitals. The workload of Polish doctors and nurses is illustrated by data from the Health at a Glance report, according to which Poland is among the countries with the lowest number of doctors and nurses per 1000 inhabitants and, at the same time, with one of the highest numbers of hospital beds per 1000 inhabitants. This overload is also clearly visible in the experiences of the patients themselves. Only 26% of the population in Poland is satisfied with the availability and quality of healthcare, compared to the OECD average of 71%. Thus, Poland occupies the last place in the entire ranking among 44 countries [27,28]. Combining all these factors—burdens on the medical professions, working circumstances across various healthcare systems, and additional burdens brought on by the COVID-19 pandemic—a picture of the struggles and obstacles that healthcare professionals deal with daily becomes clear. For this reason, it is so important to check the impact of these factors, both currently and at different stages of the pandemic, on the level of stress, mental health, or burnout among healthcare workers. The main objective of such monitoring should be to draw conclusions and build solutions for the future. This study analyzed the symptoms of health disorders, burnout, and the level of perceived stress among hospital employees throughout Poland during the prolonged COVID-19 pandemic. The obtained results are an important contribution to current knowledge about the psychological reactions of healthcare workers to working conditions during the pandemic.

## 2. Materials and Methods

### 2.1. Study Design and Participants

The main task of this study was to assess the psychophysical condition of healthcare workers in Poland. For this purpose, a cross-sectional study was designed to assess factors such as depressive symptoms, somatic symptoms, level of exhaustion, level of perceived stress, and burnout. The main criterion for inclusion was to work in the hospital, including all positions and ages. The invitations to participate in this study were addressed directly to healthcare professionals working in hospitals throughout Poland. In addition, a procedure was used in which only fully completed questionnaires were recorded, thus avoiding surveys with missing answers. Therefore, all the people who completed the task were included in this study. Overall, there were 335 healthcare workers, with a median age of 44 years. Most of the sample was female (86.3%). Among the studied groups were the following: medical staff working directly with patients (doctors, nurses, paramedics, physiotherapists), a group dedicated only to patients with COVID-19, and other hospital employees (technicians, administration, etc.).

### 2.2. Data Collection (Procedure, Instruments)

The survey was conducted among employees of the healthcare system in Poland. Medical workers (doctors, nurses, physiotherapists, paramedics, etc.) and administrative staff were invited to participate in this study. From the 16th of August 2021 to the 30th of March 2022, applications for the permission to conduct an electronic, anonymous questionnaire among employees were sent to hospital directors throughout Poland. Out of the 350 hospitals invited, 108 responded, of which 78 agreed to participate in this study. A link to the questionnaire with information about this study and a request to complete the questionnaire were sent to employees at consenting hospitals using the internal IT systems of the medical entities or by e-mail in the form of an internal newsletter.

The data collection method aimed to reach as many healthcare professionals as possible throughout Poland, including those working in hospitals, with various degrees of reference. The aim was to avoid the risk of collecting data within a closed social bubble, thus ensuring diversity and representativeness.

Participants were asked questions about the following: their gender, age, place of residence, education, occupation, presence of chronic diseases, mental health, history of SARS-CoV-2 infection, hospitalization in connection with SARS-CoV-2 infection, and work in a ward dedicated to patients with COVID-19. A question was also asked about the need to consult a psychologist in connection with the work performed.

The PSS-10 scale is used to measure perceived stress in connection with current life events; it was first developed by S. Cohen, T. Kamarck, and R. Mermelstein, and was utilized in this study in its Polish adaptation, made by Z. Juczyński and N. Ogińska-Bulik [29]. As a result of experiencing chronic stress of intense severity, serious consequences and disorders of both mental and somatic health can occur. In this context, this study aimed to identify the prevalence of people potentially in need of psychological help. The scale consisted of 10 questions that referred to the subjective assessment of one’s reactions in response to personal events and ways of dealing with them. Participants gave answers on a five-point Likert scale, from “never” to “very often”. A raw score of up to 13 was considered a low or negligible stress level; from 20 points to 40 were high scores indicating increased stress levels. The internal consistency of the scale was 0.86 Cronbach’s alpha, while the internal reliability varied between 0.84 and 0.86 Cronbach’s alpha. In Polish normalization studies, the average results were SD = 7.5 and M = 16.62, and they are higher than the average results obtained in the American sample [29].

The LBQ is designed to measure burnout in people working in professions related to helping other people and teaching; it was developed by M. Santinello and G. Altoe. The official Polish-language version made by the Psychological Tests Team of the Polish Psychological Testing Laboratory was used [9]. As a result of strain and stress in the workplace, symptoms of burnout may develop, which are also significantly related to the symptoms of depression. An important consequence of the appearance of symptoms of burnout may be the deterioration in the quality of work, including more frequent mistakes made by medics. The purpose of this questionnaire is to isolate people who are at risk. LBQ consists of 24 items describing the participant’s feelings about his or her professional work, with answers given on a 6-point Likert scale and higher scores indicating greater frequency with which feelings appear. Scores from 6 to 11 are considered low level, while scores of 25 and above indicate a high level of burnout. The LBQ assesses four aspects of burnout: psychophysical exhaustion (a dimension related to the assessment of one’s psychophysical resources); lack of involvement in customer relations (a dimension describing the quality of customer relations); the feeling of professional ineffectiveness (a dimension that refers to the assessment of one’s professional competence); and disappointment (a dimension of existential expectations). Cronbach’s alpha reliability coefficients for the individual scales were 0.77, 0.69, 0.68, and 0.85, respectively [9].

The GHQ-28 assesses the mental health of adults whose mental state may have temporarily collapsed as a result of environmental circumstances or experienced difficulties. The questionnaire was created by David Goldberg, and the Polish adaptation was completed by Z. Makowska and D. Merecz [30,31]. The use of the GHQ-28 questionnaire allows one to estimate the potential psychological consequences of functioning in conditions of prolonged stress, describe the symptoms, and determine the potential risk of mental disorders in the studied group. This questionnaire can also be used to assess the mental health impact of exposure to stressors in the workplace, in this case among hospital workers in Poland [30]. Participants give answers on a 4-point scale from “less than usual” to “much more than usual”. This questionnaire is derived from the basic version of the 60-question David Goldberg GHQ. The GHQ-28 version, in addition to the overall score, includes four scales: A—somatic symptoms; B—anxiety, insomnia; C—functional disorders; and D—symptoms of depression. An overall score of up to 16 points is considered low/no disorder, while above 28 is considered high, with many symptoms of poor health. Cronbach’s alpha ranges between 0.82 and 0.93 [31] while, in the Polish version, Cronbach’s alpha varied between 0.91 and 0.93, and the test–retest reliability index was 0.68 [30].

### 2.3. Ethics

This study was approved by the Independent Bioethics Committee for Scientific Research of the Medical University of Gdańsk by resolution NKBBN/229-123/2021 in accordance with the Declaration of Helsinki. All methods were performed in accordance with the relevant ethical guidelines and regulations. Informed consent was obtained from all the participants, and anonymity was maintained. The method of data collection was chosen in such a way that it was also impossible to identify the workplaces of the people taking part in this study.

### 2.4. Statistical Analysis

For statistical analysis of significant dependencies, the results of the PSS-10 and GFQ28, the PSS-10 and LBQ, and the GFQ28 and LBQ were correlated, and Pearson’s r was calculated. To assess the impact of individual variables on the level of perceived stress, general mental health, and burnout, data from individual tests and declarations of participants in the analyzed variables were examined. To compare the differences between the studied variables, which were due to the large disproportion in the numbers of the compared groups, the result was also verified through a Mann–Whitney U test.

## 3. Results

### 3.1. Description of the Group of Respondents

In the presented study, 335 healthcare workers between the ages of 18 and 72 took part in the study (mean age 43 years, median age 44 years). Most of the sample was female (86.3%, *n* = 289), and 13.7% (*n* = 46) were male. Most participants had attended higher education (70.2%). Among the medical workers, 37.6% had been infected with the SARS-CoV-2 virus, of which only one was hospitalized due to infection. Medical staff working directly with patients (doctors, nurses, paramedics, and physiotherapists) accounted for 58.2% (*n* = 195) of the participants, while other hospital employees (technicians, laboratory diagnosticians, etc.) accounted for 10.9% (*n* = 36), and administration employees 31% (*n* = 104). In the study group, 27.5% worked in a ward dedicated only to patients with COVID-19. Among the surveyed healthcare workers, 29% of respondents are chronically ill, 12.8% declared depression diagnosed before the pandemic, and 8.4% have anxiety disorders (Table 1).

### 3.2. Level of Perceived Stress (PSS-10 Results)

**Figure 1:**
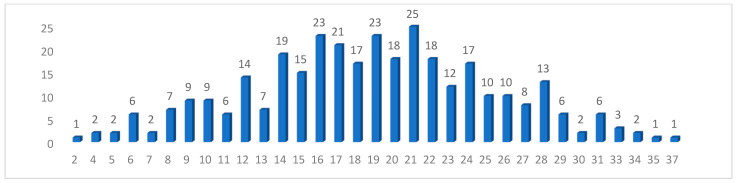
Numerical distribution of the PSS-10 results.

Overall, 19.4% (*n* = 65) of the participants had low scores on the Perceived Stress Scale, while 40.6% had average scores, and 40.0% had high scores (Figure 1). In the present study, the Cronbach alfa coefficient for the PSS-10 test was α = 0.875.

### 3.3. Burnout Level (LBQ Scores)

Overall, 9.6% (*n* = 32) of the participants had high scores on the burnout scale, 74.9% (*n* = 251) had average scores (including *n* = 54, 16.1%, at the upper limit), and 15.2% (*n* = 51) had low scores. In the present study, the Cronbach alfa coefficient for the LBQ test was α = 0.903, and for the individual subscales WP/α = 0.798, BZR/α = 0.700, SK/α = 0.690, and ROZ/α = 0.867.

The participants primarily declared some problems related to the psychophysical exhaustion at work and a weaker commitment to relationships and disappointment (although this was not a very high level), as well as a low self-efficacy (Table 2).

### 3.4. Health Assessment (GHQ-28)

Although the average scores on the individual scales did not exceed the norm, 49.6% (*n* = 166) of the participants had high scores on the GHQ-28, while 33.1% (*n* = 111) had average results, and 17.3% (*n* = 58) had low results. The participants had higher scores on somatic symptoms and lower scores on symptoms of depression (Table 3). In the present study, the Cronbach alfa coefficient for the GHQ-28 test was α = 0.950, and for the individual subscales A/α = 0.860, B/α = 0.903, C/α = 0.879, and D/α = 0.908.

### 3.5. Correlations between the Level of Perceived Stress, the Assessment of Mental Health and Burnout, and the Variables Studied

Statistically significant positive correlations were obtained between all the GHQ-28 scales and the PSS-10 scores (Table 4). Participants who scored higher on the stress scale also scored higher on all scales (somatic symptoms, anxiety and insomnia, functional disorders, and symptoms of depression) of the GHQ-28. Similarly, statistically significant positive correlations were obtained between all the LBQ scales and the PSS-10 scores. Participants who scored higher on the stress scale also scored higher on the burnout symptoms’ scales (LBQ). Statistically significant positive correlations were also obtained for the LBQ and GHQ-28 scales, with those scoring higher on the GHQ-28 scale also scoring higher on the LBQ burnout scales (Table 4).

There were two positive statistically significant compounds (Table 5). Participants reporting anxiety and depressive disorders also reported greater stress. Nine statistically significant positive correlations were also obtained (Table 5). Participants with mood disorders, depressive symptoms, and anxiety disorders also had greater somatic symptoms, a sense of anxiety, sleep problems, functional disorders, and depressive symptoms. In addition, chronically ill participants also reported greater depressive symptoms. There was no statistically significant correlation between previous health problems and burnout (Table 5).

Participants with chronic diseases reported significantly more somatic symptoms, although participants reporting depression or anxiety disorders reported significantly more somatic symptoms, anxiety and insomnia, functional disorders, and depressive symptoms (Table 6).

Access to psychological help in the workplace was reported by 34.3% (*n* = 115) of the participants, while the remainder of the healthcare workers, 65.7% (*n* = 220), either did not have access to such help or did not know about it (Table 7).

The need to consult a psychologist in connection with their work was reported by 21.5% (*n* = 72) of the participants, while 78.5% (*n* = 263) did not feel such a need (Table 7).

Statistically significant positive correlations were obtained between needing to consult a psychologist and all the other studied variables (Table 8). The participants declaring a strong need for contact with a psychologist had higher scores on all the scales of the GHQ-28 (somatic symptoms, anxiety and insomnia, general functional disorders, and symptoms of depression), LBQ (psychophysical exhaustion, lack of commitment to customer relations, a sense of lack of professional effectiveness, and disappointment), and PSS-10 (Table 8).

### 3.6. Differences between Occupational Groups

For the results of the PSS-10 (F = 1.08; *p* > 0.05) and GHQ-28 (F = 1.78; F = 0.85; F = 0.62; F = 0.54; *p* > 0.05) questionnaires, no statistically significant differences were obtained between the compared groups. For the LBQ scores, one statistically significant difference was obtained, in the “Disappointment” scale (F = 5.38; *p* < 0.01). The analyses of detailed post-group comparisons (b’Tuckey’s) indicated that there was a significant difference in the level of disappointment between groups A and E. The administrative staff indicated a higher level of burnout, especially compared to the nurses (Table 9). Due to the disproportions in the size of the groups, the results were also verified through the non-parametric Kruskal–Wallis test.

### 3.7. The Importance of Working in a COVID Ward for the PSS-10, LBQ, and GHQ-28 Test Results

In the univariate ANOVA analysis, there were no statistically significant differences between the compared groups. The fact of working in a COVID ward did not significantly differentiate the healthcare workers in terms of any of the variables studied (PSS-10, LBQ, GHQ-28) (Table 10).

Due to the disproportions in the size of the groups, the result was also verified through the non-parametric Kruskal–Wallis test.

## 4. Discussion

This study aimed to assess the mental health and burnout of healthcare workers during the one-and-a-half-year COVID-19 pandemic. In addition, we also explored the relationship between stress levels, mental health assessments, and burnout.

In this study, 21.5% of the participants were at a very high risk of burnout, experiencing mostly psychophysical exhaustion at work. Almost half of the surveyed healthcare workers experienced health disorders on a mental and physiological level. The healthcare professionals experiencing high stress also experienced significant health disorders and severe symptoms of burnout. Interestingly, the medical professionals having direct contact with COVID-19 patients experienced similar levels of stress, burnout, and overall mental health as the workers in other healthcare settings.

Numerous studies indicate that the outbreak of pandemics (e.g., SARS and MERS) exacerbates pre-existing mental health problems, both among healthcare workers [32,33,34] and the general population [35,36]. In this study, there were no significant differences between the different groups of healthcare professionals in terms of the perceived stress levels, burnout levels, and overall mental health. An interesting result was also obtained in the context of work in the wards for COVID-19 patients. The frontline healthcare workers did not differ in terms of the stress intensity, burnout levels, and general health from the healthcare workers who did not come into daily contact with infected patients. The data available in the literature can be contradictory. According to certain studies, frontline healthcare workers experience higher levels of symptoms of various mental health disorders than non-frontline healthcare workers [37,38,39,40]. Other research, such as this one, shows no difference between frontline and non-frontline personnel [41,42]. Others do not distinguish between frontline and backline medics [43,44]. Similar dilemmas have been observed during previous pandemics [45]. However, there is no doubt that healthcare workers suffer more from mental health issues than the general population [38,39]. The factors that may contribute to the disparities between the reports could be various. Cultural factors [41,46,47,48] may affect how individuals perceive and cope with stressors, while the stage of the pandemic and the restrictions in a given country can determine the level of fear and uncertainty [41,47]. Additionally, the number of infections [46] and the state of the healthcare systems can contribute to the feelings of anxiety and helplessness. Access to psychological help [41] and social support [47] is crucial in mitigating the negative effects on mental well-being during these challenging times. Different variables appeared to be the reason for the lack of differences in the rate of psychological distress in both groups. On the one hand, the frontline healthcare workers were in direct contact with COVID-19 patients, and their worry about infecting their loved ones was one of the most common sources of recurring anxiety [41,49]. On the other hand, the non-frontline healthcare workers could also experience psychological distress due to their concerns about potential exposure to the virus in their workplace and the overall uncertainty surrounding the pandemic. Then again, it appears that the frontline healthcare workers received greater general social support [47,48], which the administrative employees may not have experienced. Subsequently, as a consequence of the widespread shortages, personal protective equipment, the availability of which had a significant impact on mental well-being [41,47], may have been unavailable for the employees of other hospital departments [42]. Furthermore, while working at a hospital, the administrative staff, like the rest of the general population, might have encountered a lack of adequate medical information, understanding of the situation, or preparation for such circumstances [42]. These variables may have contributed to a similar level of psychological distress in both groups, despite their differing levels of exposure to COVID-19 patients.

In the present study, after a year and a half of the pandemic, a high level of stress intensity was observed among healthcare workers, with 40% of the participants reporting a high stress intensity. A multicenter study similarly found that the stress levels among healthcare workers increased during the pandemic, such that, initially, 53.8% of the participants experienced high levels of stress, which later increased to 61.6% [50]. A study assessing stress levels based on the cortisol levels in hair also found elevated hair cortisol levels in 40% of the healthcare professionals assessed [51]. Slightly lower scores were obtained in a study conducted in Pakistan, whereby 33.9% of the sample of medics experienced high stress [36]. Among the medics experiencing high levels of stress, we should recognize that they were mainly young people, people with less work experience, people with previous mental health problems, and females [50,52].

The level of burnout is currently higher than before the pandemic [33]. In this study, 9.6% of the participants obtained high scores on the burnout scale, while 74.9% obtained results indicating already existing problems, including 21.5% at a very high risk of burnout. Depending on the tool and assessment criteria used, as well as the variables taken into account, such as occupation, high risk or burnout can affect from 12.0% [51] to 61.7% of healthcare workers [18,21,52,53,54,55,56]. In terms of burnout symptoms, medics primarily experience a psychophysical exhaustion at work, a deterioration in relations with the patient, and a lack of effectiveness, consistent with a study carried out in Italy [57]. Other studies also indicate that the most frequently experienced symptom of burnout is exhaustion [33]; yet, in the study conducted in 2018 among Polish nurses, the lack of involvement in relations with the patients showed the greatest increase, among burnout symptoms, while disappointment rated the lowest [58].

In terms of general health (GHQ-28), almost half of the participants scored high, suggesting that almost half of the surveyed healthcare workers experienced health disorders on a mental and physiological level. In another Polish study, conducted at the beginning of the pandemic, 60% of the medics surveyed reported health disorders, [59] while another study conducted during the later stages of the pandemic found fewer health effects (39.3% obtained results pointing to health disruption) [60]. Studies generally suggest higher levels of symptoms of the GHQ-28 in medical professions [35,59]. The participants obtained the highest results in terms of somatic symptoms and the lowest in the range of depression symptoms. Iranian physicians scored highest on the scale of anxiety symptoms and insomnia, while their depression scores were the lowest [61].

In the present study, the healthcare professionals experiencing high stress (PSS-10) also experienced significant health disorders (GHQ-28). It may be that medics feel a strong anxiety, complain of insomnia, indicate more somatic symptoms, as well as symptoms of depression, and declare more functional disorders. In addition, the participants declaring severe stress were also found to experience severe symptoms of burnout (LBQ). Among them, they reported primarily a sense of psychophysical exhaustion, ineffectiveness, and disappointment, as well as a low level of involvement in relationships with patients (although this was reported slightly less). All the participants complaining about the state of their health (GHQ-28) in its various aspects also declared symptoms of burnout—a sense of physical exhaustion, lack of involvement in their contact with patients, and a sense of ineffectiveness and disappointment with everyday life. Emotional exhaustion affects 46% of medics, and between 35% and 60% of healthcare workers experience a variety of somatic symptoms. In addition, research indicates a link between severe stress, depression, and insomnia [62].

Among the medics with pre-existing health problems (i.e., problems experienced before the pandemic), the participants declaring anxiety and depressive disorders also indicated an increase in experienced stress. In addition, they also indicated somatic symptoms, a sense of anxiety, sleep problems, functional disorders, and depressive symptoms. The chronically ill people also indicated more depressive symptoms.

Access to psychological help in the workplace was declared by 34.3% of the healthcare workers, while the need to consult a psychologist in connection with their work was declared by 21.5%; this is consistent with other studies suggesting that healthcare workers rarely seek psychological help [1,2,52,63]. More optimistic results were obtained in the survey of healthcare workers that had been conducted in Poland before the pandemic (2018/2019). At that time, 47% of the healthcare workers surveyed declared access to psychological help in the workplace, and 56.4% declared their willingness to use psychological help [64]. The participants declaring a strong need for contact with a psychologist obtained high scores on all the scales of the GHQ-28 test (somatic symptoms, anxiety, and insomnia, general functional disorders, and symptoms of depression), LBQ (psychophysical exhaustion, lack of involvement in customer relations, a sense of lack of professional effectiveness, and disappointment), and PSS-10; therefore, at least 21% of the healthcare professionals surveyed experienced serious mental health disorders.

Participants with burnout may experience exhaustion, sleep problems, and depersonalization, as well as cognitive, memory, and attention disorders [7,10]; in turn, these can translate into a deterioration in the quality of work or in making more mistakes. Lowering the quality of patient care [20,51,62] also translates into lower patient satisfaction [7,18,21]. In a comparative study of burnout among nurses, the Polish nurses had a higher level of burnout compared to the nurses from Denmark [65]. Many studies show the co-occurrence of burnout and depression [2,20,21,53,66], symptoms of anxiety [61], and even PTSD [66]. In addition, it is estimated that between 5% and 10% of medics experience suicidal thoughts [2,67], and the prevalence of suicide among medics is higher than among the general population [67], especially among nurses [2,57,67].

The results of this study indicate that the mental conditions of healthcare workers are poor and very worrying, and not only in Poland [34]. Taking into account working conditions both before and during the COVID-19 pandemic, a marked increase in the intensity of stress, symptoms of health disorders, or symptoms of burnout among healthcare workers during the COVID-19 pandemic is not surprising; however, initial data from before the pandemic were also worrying.

Considering the number of people experiencing health disorders, severe stress, and burnout, the number of people declaring the need for psychological consultations, and the number of people having access to psychological care in the workplace, it is clear that some medics do not see the need for psychological help. It is also clear that healthcare workers are in poor shape and, unfortunately, are not receiving the care and specialist support they need for their mental health.

Decisive actions must be taken to improve labor standards, such as rest conditions, chill rooms, and daycare for children. The introduction of mandatory training for managers surrounding human resources management, communication with subordinates, team building, providing feedback, and non-violent communication is required in order to support them in creating a respectful workplace environment. In addition, it is crucial to establish support systems within healthcare organizations that prioritize the well-being of healthcare professionals. It is critical to provide dedicated psychologists for healthcare staff, with whom they do not cooperate in the context of patients. These psychologists can offer confidential counseling sessions to healthcare staff, allowing them to address any work-related stress or emotional challenges they may be facing. Additionally, implementing regular mental health check-ins and creating support groups within the workplace can further promote a positive and supportive environment for healthcare professionals. It is important to provide workshops on building resources for dealing with difficult situations and on how to take care of one’s own mental health (relaxation, mindfulness, etc.). Furthermore, due to the lack of personnel, developing initiatives that encourage young individuals to pursue careers in the medical field, such as scholarships, mentorship programs, and career fairs, is important. By taking proactive measures now, we can ensure a resilient healthcare system that is better equipped for future crisis events.

### Limitations

Despite significant findings, the authors encountered several difficulties. Unfortunately, as in many studies, it was not possible to avoid significant discrepancies in the number of women and men studied. Significant discrepancies in the size of the studied groups were obtained between individual occupational groups, e.g., unquestionably more nurses than doctors. The method of data collection, the aim of which was to reach as many employees of Polish hospitals as possible and to avoid collecting data within a so-called “social bubble”, also had some limitations. Unfortunately, after forwarding the link to this study to the appropriate units of the hospitals, the researchers no longer had control over what happened with the invitation. This could have had a significant impact on the amount of data finally collected, despite the wide reach to hospitals. Due to the data collection method used, we do not have data on how many people received an invitation to participate in this study, and, thus, it is not possible to estimate the response rate.

## 5. Conclusions

The current mental health of healthcare workers is concerning. Many healthcare workers, as a result of the characteristics of their profession and the additional burdens resulting from working in pandemic conditions, experienced high levels of stress, deterioration of mental health, or increased symptoms of burnout. In the studied group of healthcare workers, no differences were observed in the symptoms of mental health disorders between the frontline workers and the other employees of Polish hospitals. In connection with the above, it is worth paying special attention to two directions of research. First, the mental health of healthcare workers should continue to be monitored to see how long the impact of the pandemic on mental health will last. Second, we need to take a closer look at the factors influencing the mental health of healthcare workers during the pandemic, and at what variables affect the well-being of frontline and non-frontline healthcare workers.

## Figures and Tables

**Table 1:** Demographic data of the respondents.

Characteristic	N	%
Total	335	
Gender		
Female	289	86.3%
Male	46	13.7%
Education		
higher education	235	70.2%
bachelor’s, engineer	50	14.9%
secondary education	50	14.9%
Occupational group		
medical	195	58.2%
nurse	109	32.5%
physician	46	13.7%
other medical workers	40	11.9%
other specialists	36	10.9%
administration	140	41.8%
Health status		
chronic diseases	97	29%
depression	43	12.8%
anxiety	28	8.4%
COVID-19 infection	126	37.6%
COVID-19 ward	92	27.5%

**Table 2:** Numerical distribution of the results obtained in the LBQ.

	M	SD	D	Min	Max
Psychophysical exhaustion	19.55	6.753	13	6	35
Lack of engagement with customers	17.71	5.889	12	7	36
Feeling of professional ineffectiveness	13.86	5.058	9	6	33
Disappointment	17.07	7.292	11	6	35
Total	68.19	20.357	70	30	134

M—mean, SD—standard deviation, and D—dominant.

**Table 3:** Numerical distribution of the results obtained in the GHQ-28.

	M	SD	D	Min	Max
A: somatic symptoms	9.23	4.626	8	0	21
B: anxiety, insomnia	9.13	5.066	7	0	21
C: functional disorders	8.81	3.284	7	3	21
D: symptoms of depression	3.31	4.489	0	0	21
Total	30.47	14.905	17	5	84

M—mean, SD—standard deviation, and D—dominant.

**Table 4:** Values of the r-Pearson correlation coefficients of the PSS-10, LBQ, and GHQ-28 results.

	A Somatic Symptoms	B Anxiety, Insomnia	C Functional Disorders	D Symptoms of Depression	Stress
Psychophysical exhaustion	0.517 ***	0.560 ***	0.548 ***	0.474 ***	0.612 ***
Lack of engagement with customers	0.308 ***	0.256 ***	0.294 ***	0.206 ***	0.308 ***
Feeling of professional ineffectiveness	0.393 ***	0.466 ***	0.566 ***	0.488 ***	0.579 ***
Disappointment	0.425 ***	0.470 ***	0.485 ***	0.419 ***	0.510 ***
Stress	0.632 ***	0.766 ***	0.650 **	0.627 ***	-

*** *p* < 0.01; ** *p* < 0.05.

**Table 5:** Correlation coefficients of PSS-10, GHQ-28, and health problems.

	PSS-10	GHQ-28			
Health Problems	Stress	Somatic Symptoms	Anxiety, Insomnia	Functional Disorders	Symptoms of Depression
Chronic illness	0.033	0.073	0.085	0.093	0.119 **
Depression	0.128 **	0.122 **	0.119 **	0.139 **	0.189 ***
Anxiety disorders	0.177 ***	0.188 ***	0.184 **	0.165 **	0.143 **
Other mental illnesses	0.070	0.061	0.062	0.015	0.027

*** *p* < 0.01; ** *p* < 0.05.

**Table 6:** Comparison of the average GHQ-28 scores in the people with health problems.

	Chronic Illness		Lack				
	M	SD	M	SD	t	Df	d
Symptoms of depression	4.14	5.264	2.97	4.097	1.974 **	145	0.271
	Depression/Mood disorders	Lack					
	M	SD	M	SD	t	Df	d
Somatic symptoms	10.70	4.950	9.01	4.546	2.246 **	333	0.371
Anxiety, insomnia	10.70	5.092	8.90	5.029	2.188 **	333	0.358
Functional disorders	10.00	3.703	8.64	3.188	2.292 **	333	0.427
Symptoms of depression	5.51	6.181	2.98	4.099	2.600 **	333	0.578
	Anxiety disorders	Lack					
	M	SD	M	SD	t	Df	d
Somatic symptoms	12.11	5.202	8.96	4.488	3.499 ***	333	0.677
Anxiety, insomnia	12.21	4.740	8.85	5.008	3.421 ***	333	0.661
Functional disorders	10.61	4.289	8.65	3.135	2.360 **	333	0.588
Symptoms of depression	5.43	6.735	3.11	4.190	2.634 ***	333	0.513

M—mean, SD—standard deviation, t—test value, Df—degrees of freedom, and d—Cohen effect size. *** *p* < 0.01; ** *p* < 0.05.

**Table 7:** Access and need for psychological help.

	Definitely Not	Rather Not	It is Hard to Say	Probably Yes	Definitely Yes
Do you have access to consultation with a psychologist in the workplace?	79	80	61	70	45
In connection with your work, do you feel the need to consult a psychologist?	76	125	62	52	20

**Table 8:** Values of the r-Pearson correlation coefficients of the GHQ-28, LBQ, and PSS-10 results and the need for psychological consultation.

GHQ-28	A Somatic Symptoms	B Anxiety, Insomnia	C Functional Disorders	D Symptoms of Depression	PSS-10 Stress
In connection with your work, do you feel the need to consult a psychologist?	0.147 ***	0.174 ***	0.151 ***	0.180 ***	0.182 ***
LBQ	Psychophysical exhaustion	Lack of engagement with customers	Feeling of professional ineffectiveness	Disappointment	
In connection with your work, do you feel the need to consult a psychologist?	0.262 ***	0.193 ***	0.141 ***	0.166 ***	

*** *p* < 0.01.

**Table 9:** The ANOVA univariate comparisons of the mean scores for the PSS-10, LBQ, and GHQ-28.

	Group A		Group B		Group C		Group D		Group E		
	M	SD	M	SD	M	SD	M	SD	M	SD	F
PSS-10	18.74	6.76	18.37	7.23	19.48	5.87	17	5.73	19.38	6.17	1.08
LBQ PE	18.83	6.33	21.65	7.2	20.08	6.81	18.61	6.63	19.51	6.92	1.67
LICR	17.14	5.81	17.8	5.55	18.6	5.73	17.28	6.23	18.09	6.09	0.64
PI	13.77	4.82	14.93	5.56	13.95	4.53	12.39	3.88	13.96	5.56	1.31
DIS	15.1	6.43	15.63	7.32	18.63	6.64	17	7.3	19.2	7.76	5.38 ***
GHQ-28 A	9.64	5.06	8.02	4.1	8.95	4.1	8.28	4.07	9.76	4.67	1.78
B	8.98	5.26	8.74	5.02	9.68	5.04	8.06	4.39	9.62	5.12	0.85
C	8.75	3.12	8.7	3.61	8.63	2.63	8.28	3.12	9.18	3.59	0.62
D	2.95	4.03	3.2	5.05	3.38	3.83	3	4.39	3.81	4.96	0.54

*** *p* < 0.01. M—mean, SD—standard deviation, and F—test value; Group A—nurses, Group B—physicians, Group C—other medical workers, Group D—other specialists, and Group E—administration; LBQ: PE—psychophysical exhaustion, LICR—lack of involvement in customer relations, PI—the feeling of professional ineffectiveness, and DIS—disappointment.

**Table 10:** The Univariate ANOVA for the mean PSS-10, LBQ, and GHQ-28 questionnaire scores among the people working in a COVID ward.

			Yes, I Work		Worked		I Did Not Work		Does Not Apply to Me		
		Scale	M	SD	M	SD	M	SD	M	SD	F
Group A	PSS-10		17.77	7.37	17.57	6.88	19.72	6.46	14.33	2.52	1.21
Group B	PSS-10		19.64	6.31	15.75	5.75	18.65	8.11	18.00	0.00	0.71
Group A	LBQ	PE	17.61	6.64	18.50	6.62	19.82	6.04	12.67	3.51	1.89
		LICR	16.61	5.01	18.71	6.90	17.25	5.99	13.00	4.00	0.94
		PI	13.58	5.76	12.29	2.56	14.34	4.68	11.00	4.58	1.08
		DIS	13.35	6.30	15.93	6.62	16.13	6.30	8.33	3.22	2.57
Group B	LBQ	PE	22.64	6.95	22.38	6.28	21.23	7.80	16.00	0.00	0.32
		LICR	20.36	5.84	17.88	5.33	16.69	5.45	18.00	0.00	1.14
		PI	15.64	5.03	12.25	4.98	15.50	5.98	14.00	0.00	0.77
		DIS	17.27	6.92	13.88	6.66	15.58	7.90	13.00	0.00	0.36
Group A	GHQ-28	A	9.90	5.74	11.21	6.52	9.28	4.34	7.00	3.61	0.85
		B	9.03	5.75	9.86	5.48	8.92	5.07	5.67	2.89	0.52
		C	8.42	3.52	8.71	3.36	9.02	2.94	7.00	0.00	0.57
		D	2.81	4.44	2.79	4.37	3.16	3.87	1.00	1.73	0.31
Group B	GHQ-28	A	7.36	3.85	7.25	3.66	8.54	4.46	8.00	0.00	0.31
		B	8.45	4.44	8.25	4.89	9.00	5.53	9.00	0.00	0.06
		C	8.91	2.55	8.50	3.38	8.62	4.20	10.00	0.00	0.06
		D	3.18	2.79	1.38	3.11	3.85	6.20	1.00	0.00	0.54

M—mean, SD—standard deviation, and F—test value; Group A—nurses, Group B—physicians.

## Data Availability

The datasets generated and analyzed during the current study are available from the corresponding author on reasonable request.

## References

[1] Søvold L.E., Naslund J.A., Kousoulis A.A., Saxena S., Qoronfleh M.W., Grobler C., Münter L. (2021). Prioritizing the Mental Health and Well-Being of Healthcare Workers: An Urgent Global Public Health Priority. Front. Public Health.

[2] Zisook S., Doran N., Mortali M., Hoffman L., Downs N., Davidson J., Fergerson B., Rubanovich C.K., Shapiro D., Tai-Seale M. (2022). Relationship between burnout and Major Depressive Disorder in health professionals: A HEAR report. J. Affect. Disord..

[3] Liang Y., Wu K., Zhou Y., Huang X., Zhou Y., Zhang X.Y. (2020). Mental health in frontline medical workers during the 2019 novel coronavirus disease epidemic in china: A comparison with the general population. Int. J. Environ. Res. Public Health.

[4] Zhang X., Zhao K., Zhang G., Feng R., Chen J., Xu D., Liu X., Ngoubene-Atioky A.J., Huang H., Liu Y. (2020). Occupational stress and mental health: A comparison between frontline medical staff and non-frontline medical staff during the 2019 novel coronavirus disease outbreak. Front. Psychiatry.

[5] Han S., Choi S., Cho S., Lee J., Yun J. (2021). Associations between the working experiences at frontline of covid-19 pandemic and mental health of korean public health doctors. BMC Psychiatry.

[6] Cocchiara R.A., Peruzzo M., Mannocci A., Ottolenghi L., Villari P., Polimeni A., Guerra F., La Torre G. (2019). The Use of Yoga to Manage Stress and Burnout in Healthcare Workers: A Systematic Review. J. Clin. Med..

[7] Khammissa R.A., Nemutandani S., Shangase S.L., Feller G., Lemmer J., Feller L. (2022). The burnout construct with reference to healthcare providers: A narrative review. SAGE Open Med..

[8] Białek K., Sadowski M. (2019). Level of stress and strategies used to cope with stress by physicians working in intensive care units. Anaesthesiol. Intensive Ther..

[9] Santinello M. (2014). LBQ Kwestionariusz Wypalenia Zawodowego Link. Podręcznik.

[10] Peterson U., Demerouti E., Bergström G., Samuelsson M., Asberg M., Nygren A. (2008). Burnout and physical and mental health among Swedish healthcare workers. J. Adv. Nurs..

[11] Jaracz M., Rosiak I., Bertrand-Bucińska A., Jaskulski M., Nieżurawska J., Borkowska A. (2017). Affective temperament, job stress and professional burnout in nurses and civil servants. PLoS ONE.

[12] Owoc J., Mańczak M., Tombarkiewicz M., Olszewski R. (2021). Burnout, well-being, and self-reported medical errors among physicians. Pol. Arch. Intern. Med..

[13] Nowakowska I., Rasińska R., Głowacka M.D. (2016). The influence of factors of work environment and burnout syndrome on self-efficacy of medical staff. Ann. Agric. Environ. Med. AAEM.

[14] Kwiatkowska-Ciotucha D., Załuska U., Kozyra C. (2021). The Perception of Occupation by Hospital Nurses in Poland and Germany in Terms of the Risk of Excessive Stress and Burnout as Well as Possible Coping and Preventive Solutions. Int. J. Environ. Res. Public Health.

[15] World Health Organisation. https://covid19.who.int/.

[16] The Official Website of the Government of Poland. www.gov.pl.

[17] Wojczyk M., Niewiadomska E., Kowalska M. (2023). The Incidence Proportion of SARS-CoV-2 Infections and the Percentage of Deaths among Infected Healthcare Workers in Poland. J. Clin. Med..

[18] Park S.Y., Cheong H.S., Kwon K.T., Sohn K.M., Heo S.T., Lee S., Chung U.S., Lee S.H. (2023). Guidelines for Infection Control and Burnout Prevention in Healthcare Workers Responding to COVID-19. Infect. Chemother..

[19] Szwamel K., Kaczorowska A., Lepsy E., Mroczek A., Golachowska M., Mazur E., Panczyk M. (2022). Predictors of the Occupational Burnout of Healthcare Workers in Poland during the COVID-19 Pandemic: A Cross-Sectional Study. Int. J. Environ. Res. Public Health.

[20] Ulfa M., Azuma M., Steiner A. (2022). Burnout status of healthcare workers in the world during the peak period of the COVID-19 pandemic. Front. Psychol..

[21] Hajebi A., Abbasinejad M., Zafar M., Hajebi A., Taremian F. (2022). Mental Health, Burnout, and Job Stressors Among Healthcare Workers During the COVID-19 Pandemic in Iran: A Cross-Sectional Survey. Front. Psychiatry.

[22] Ochronne śluzy z PWr w szpitalach w Legnicy i Wrocławiu. https://pwr.edu.pl/uczelnia/aktualnosci/ochronne-sluzy-z-pwr-w-szpitalach-w-legnicy-i-wroclawiu-11975.html.

[23] “Brawa Dla Was”. Podziękowania i Oklaski dla Walczących z Pandemią w Polskich Miastach. https://tvn24.pl/polska/koronawirus-w-polsce-brawa-dla-was-oklaski-dla-lekarzy-i-sluzb-medycznych-4546955.

[24] Rośnie Hejt Wobec Medyków w Czasie Koronawirusa. Teraz to “Łajza, co Zarazę Przenosi”. https://lodz.wyborcza.pl/lodz/7,35136,25880123,klaskalismy-dla-medyka-bo-walczy-z-koronawiursem-teraz-to.html.

[25] Zatrzymaj Hejt. https://nszzp.pl/aktualnosci/zatrzymaj-hejt-czyli-wspierajmedyka/.

[26] Apuke O.D., Omar B. (2020). Modelling the antecedent factors that affect online fake news sharing on COVID-19: The moderating role of fake news knowledge. Health Educ. Res..

[27] OECD (2019). Health at a Glance 2019: OECD Indicators.

[28] OECD (2021). Health at a Glance 2021: OECD Indicators.

[29] Juczyński Z., Ogińska-Bulik N. (2012). Narzędzia Pomiaru Stresu i Radzenia Sobie ze Stresem.

[30] Makowska Z., Merecz D. (2001). Ocena Zdrowia Psychicznego na Podstawie Badań Kwestionariuszami Davida Goldberga Polska Adaptacja Kwestionariuszy Ogólnego Stanu Zdrowia Davida Goldberga: GHQ-12 i GHQ-28.

[31] Goldberg D., Williams P. (2001). Podręcznik dla Użytkowników Kwestionariusza Ogólnego Stanu Zdrowia.

[32] Ciułkowicz M., Maciaszek J., Misiak B., Pałȩga A., Rymaszewska J., Szcześniak D.M. (2021). Coping Strategies and Psychopathological Responses Among Medical and Non-medical Professionals—A Cross-Sectional Online Survey. Front. Psychiatry.

[33] Akerstrom M., Sengpiel V., Hadžibajramović E., Carlsson Y., Graner S., Andersson O., Jonsson M., Naurin E., Veje M., Wessberg A. (2023). The COPE Staff study: Study description and initial report regarding job satisfaction, work-life conflicts, stress, and burnout among Swedish maternal and neonatal healthcare workers during the COVID-19 pandemic. Int. J. Gynaecol. Obstet..

[34] Lee B.E.C., Ling M., Boyd L., Olsson C., Sheen J. (2023). The prevalence of probable mental health disorders among hospital healthcare workers during COVID-19: A systematic review and meta-analysis. J. Affect. Disord..

[35] Babicki M., Szewczykowska I., Mastalerz-Migas A. (2021). Mental Health in the Era of the Second Wave of SARS-CoV-2: A Cross-Sectional Study Based on an Online Survey among Online Respondents in Poland. Int. J. Environ. Res. Public Health.

[36] Nadeem M.U., Kulich S.J., Bokhari I.H. (2023). The assessment and validation of the depression, anxiety, and stress scale (DASS-21) among frontline doctors in Pakistan during fifth wave of COVID-19. Front. Public Health.

[37] Tabano S., Tassi L., Cannone M.G., Brescia G., Gaudioso G., Ferrara M., Colapietro P., Fontana L., Miozzo M.R., Croci G.A. (2023). Mental health and the effects on methylation of stress-related genes in front-line versus other health care professionals during the second wave of COVID-19 pandemic: An Italian pilot study. Eur. Arch. Psychiatry Clin. Neurosci..

[38] Alshekaili M., Hassan W., Al Said N., Al Sulaimani F., Jayapal S.K., Al-Mawali A., Chan M.F., Mahadevan S., Al-Adawi S. (2020). Factors associated with mental health outcomes across healthcare settings in Oman during COVID-19: Frontline versus non-frontline healthcare workers. BMJ Open.

[39] Yan X.G., Sun H.Y., Yoo B.W. (2023). A Cross-Sectional Study on the Psychological Changes of Medical Personnel in Hospitals Who Experienced Special COVID-19 Situations. Inq. A J. Med. Care Organ. Provis. Financ..

[40] Chhablani N., Choudhari S.G. (2022). Behind the Frontline: A Review on the Impact of COVID-19 Pandemic on Healthcare Workers. Cureus.

[41] Czepiel D., Hoek H.W., van der Markt A., Rutten B.P.F., Veling W., Schirmbeck F., Mascayano F., Susser E.S., van der Ven E. (2022). The Association Between Exposure to COVID-19 and Mental Health Outcomes Among Healthcare Workers. Front. Public Health.

[42] Zhao Y.J., Xing X., Tian T., Wang Q., Liang S., Wang Z., Cheung T., Su Z., Tang Y.L., Ng C.H. (2022). Post COVID-19 mental health symptoms and quality of life among COVID-19 frontline clinicians: A comparative study using propensity score matching approach. Transl. Psychiatry.

[43] Abid A., Shahzad H., Khan H.A., Piryani S., Khan A.R., Rabbani F. (2022). Perceived risk and distress related to COVID-19 in healthcare versus non-healthcare workers of Pakistan: A cross-sectional study. Hum. Resour. Health.

[44] Rosenström T., Tuisku K., Suvisaari J., Pukkala E., Junttila K., Haravuori H., Elovainio M., Haapa T., Jylhä P., Laukkala T. (2022). Healthcare workers’ heterogeneous mental-health responses to prolonging COVID-19 pandemic: A full year of monthly follow up in Finland. BMC Psychiatry.

[45] Brooks S.K., Dunn R., Amlôt R., Rubin G.J., Greenberg N. (2018). A Systematic, Thematic Review of Social and Occupational Factors Associated with Psychological Outcomes in Healthcare Employees During an Infectious Disease Outbreak. J. Occup. Environ. Med..

[46] Rosales Vaca K.M., Cruz Barrientos O.I., Girón López S., Noriega S., More Árias A., Guariente S.M.M., Zazula R. (2022). Mental health of healthcare workers of Latin American countries: A review of studies published during the first year of COVID-19 pandemic. Psychiatry Res..

[47] De Kock J.H., Latham H.A., Leslie S.J., Grindle M., Munoz S.A., Ellis L., Polson R., O’Malley C.M. (2021). A rapid review of the impact of COVID-19 on the mental health of healthcare workers: Implications for supporting psychological well-being. BMC Public Health.

[48] Pappa S., Chen J., Barnett J., Chang A., Dong R.K., Xu W., Yin A., Chen B.Z., Delios A.Y., Chen R.Z. (2022). A systematic review and meta-analysis of the mental health symptoms during the COVID-19 pandemic in Southeast Asia. Psychiatry Clin. Neurosci..

[49] Rathod S., Pallikadavath S., Young A.H., Graves L., Rahman M.M., Brooks A., Soomro M., Rathod P., Phiri P. (2020). Psychological impact of COVID-19 pandemic: Protocol and results of first three weeks from an international cross-section survey—Focus on health professionals. J. Affect. Disord. Rep..

[50] Xiong N.N., Fan T.T., Leonhart R., Fritzsche K., Liu Q., Luo L., Stein B., Waller C., Huang M., Müller M.M. (2022). Workplace factors can predict the stress levels of healthcare workers during the COVID-19 pandemic: First interim results of a multicenter follow-up study. Front. Public Health.

[51] Ibar C., Fortuna F., Gonzalez D., Jamardo J., Jacobsen D., Pugliese L., Giraudo L., Ceres V., Mendoza C., Repetto E.M. (2021). Evaluation of stress, burnout and hair cortisol levels in health workers at a University Hospital during COVID-19 pandemic. Psychoneuroendocrinology.

[52] Briciu V., Leucuta D.C., Tőkés G.E., Colcear D. (2023). Burnout, Depression, and Job Stress Factors in Healthcare Workers of a Romanian COVID-19 Dedicated Hospital, after Two Pandemic Years. Int. J. Environ. Res. Public Health.

[53] Pan S.J., Qian W.Y., Yang Y.P., Zhang M.X., Hu X.M., Chen H.X., Tung T.H. (2022). Evaluation of burnout among stay-behind healthcare workers during the current Omicron wave of COVID-19 in Taizhou, China. Front. Psychiatry.

[54] Yang C., Wang X., Zhang X., Liu W., Wang C. (2023). Burnout and associative emotional status and coping style of healthcare workers in COVID-19 epidemic control: A cross-sectional study. Front. Public Health.

[55] Choi Y.E., Lee S.H., Kim Y.J., Lee J.G., Yi Y.H., Tak Y.J., Kim G.L., Ra Y.J., Lee S.Y., Cho Y.H. (2023). Burnout in healthcare workers in COVID-19-dedicated hospitals. J. Public Health.

[56] Burrowes S.A.B., Casey S.M., Pierre-Joseph N., Talbot S.G., Hall T., Christian-Brathwaite N., Del-Carmen M., Garofalo C., Lundberg B., Mehta P.K. (2023). COVID-19 pandemic impacts on mental health, burnout, and longevity in the workplace among healthcare workers: A mixed methods study. J. Interprofessional Educ. Pract..

[57] Epifanio M.S., La Grutta S., Piombo M.A., Riolo M., Spicuzza V., Franco M., Mancini G., De Pascalis L., Trombini E., Andrei F. (2023). Hopelessness and burnout in Italian healthcare workers during COVID-19 pandemic: The mediating role of trait emotional intelligence. Front. Psychol..

[58] Załuski M., Makara-Studzińska M. (2019). Wzajemne relacje między wypaleniem zawodowym, pracą emocjonalną i zaangażowaniem w pracę u pracowników ochrony zdrowia [The reciprocal relationship between occupational burnout, emotional labor and work engagement in healthcare specialists]. Med. Pr..

[59] Maciaszek J., Ciulkowicz M., Misiak B., Szczesniak D., Luc D., Wieczorek T., Fila-Witecka K., Gawlowski P., Rymaszewska J. (2020). Mental Health of Medical and Non-Medical Professionals during the Peak of the COVID-19 Pandemic: A Cross-Sectional Nationwide Study. J. Clin. Med..

[60] Kolivand P., Hosseindoost S., Kolivand Z., Gharaylou Z. (2023). Psychosocial impact of COVID-19 2 years after outbreak on mental health of medical workers in Iran. Middle East Curr. Psychiatry Ain Shams Univ..

[61] Babamiri M., Bashirian S., Khazaei S., Sohrabi M.S., Heidarimoghadam R., Mortezapoor A., Zareian S. (2022). Burnout and Mental Health of COVID-19 Frontline Healthcare Workers: Results from an Online Survey. Iran. J. Psychiatry.

[62] Jiang C., Jiang W., Yue Y., Li L., Sun T., Chen G., Xu W., Shah S.M., Liu X., Chen S. (2023). The trends of psychosomatic symptoms and perceived stress among healthcare workers during the COVID-19 pandemic in China: Four cross-sectional nationwide surveys, 2020-2023. Psychiatry Res..

[63] González Baltazara R., Hidalgo Santacruza G., León Cortésa S. (2015). Quality of work life and mental health in primary care physicians. Procedia Manuf..

[64] Witczak-Błoszyk K., Krysińska K., Andriessen K., Stańdo J., Czabański A. (2022). Work-Related Suicide Exposure, Occupational Burnout, and Coping in Emergency Medical Services Personnel in Poland. Int. J. Environ. Res. Public Health.

[65] Shoji K., Lesnierowska M., Smoktunowicz E., Bock J., Luszczynska A., Benight C.C., Cieslak R. (2015). What Comes First, Job Burnout or Secondary Traumatic Stress? Findings from Two Longitudinal Studies from the U.S. and Poland. PLoS ONE.

[66] Giusti E.M., Veronesi G., Callegari C., Borchini R., Castelnuovo G., Gianfagna F., Iacoviello L., Ferrario M.M. (2023). Pre-pandemic burnout and its changes during the COVID-19 outbreak as predictors of mental health of healthcare workers: A lesson to be learned. Psychiatry Res..

[67] Martínez-Arriaga R.J., Dominguez-Rodriguez A., Herdoiza-Arroyo P.E., Robles-Garcia R., de la Rosa-Gómez A., Figueroa González J.A., Muñoz Anacona Y.A. (2023). Suicide risk and associated factors in healthcare workers seeking psychological support during COVID-19: A cross-sectional study. Psychol. Health Med..

